# Compulsory Medication of Prostitutes by the State

**Published:** 1877-03

**Authors:** 


### Compulsory Medication of Prostitutes by the State.

Of the registered women in Paris, those living in the *maisons tolerees* are examined once a week, while those who live in private apartments are examined only twice a month. "But," says Dr. Mireur, who expresses the opinion of many eminent French physicians, "in the eyes of the majority of hygienists and syphiliographers, such examinations as those which public women actually undergo are incapable of affording all the guarantees which we are entitled to expect from a similar measure. In fact, while these women are examined twice a month, or even once a week, if contagious sores are developed a short time after an examination, such women may transmit their poison to a great number of men before the next examination proving them to be diseased results in their being sent to hospital for treatment. M. Ricord thinks that prostitutes ought to be examined at least every third day; M. Ratier and M. Sandouville, every fourth day; M. Davilla, M. Langlebert, and many others, twice a week; M. Lancereaux, *every day*."

While of opioion that, in respect to public women *free from syphilitic infection*, two examinations a week ought to be, not absolutely, but within the limits of reasonable precaution, a guarantee against disease, M. Langlebert asks, "But would they be so in respect to women affected with syphilitic diathesis?" and he replies, "Certaiuly not; and here it behoves us to say with M. Alfred Fournier, that 'the knowledge of the contagious character of secondary syphilis opens a new era in prophylaxy, and exacts more extensive guarantees.'" M. Langlebert goes on to say that a woman leaving the hospital after having been treated for syphilis ought to be subject to a *special supervision*, involving an introspective examination every day during a period of eighteen months or two years after she is discharged from the hospital—that is to say, during the ordinary time within which, as sequences of the primary disease, simptoms of secondary syphilis are produced or renewed(?). By way of comment on the proposal here described, Dr. Mireus observes—"Until a new prophylactic system posessing real efficacy shall be adopted, the reform advocated by M. Langlebert is indispensible; and so long as it is not put into execution, we do not hesitate to affirm that registered prostitution, as well as clandestine, will continue a perpetual source of contamination." An examination every day—especially if during a period of two years—is indeed, as M. Langlebert admits, "*beaucoup*," or a good deal to ask; "but," he adds, philosophically, "he who would achieve the end must assent to the means."

Now, when it is considered how large is the proportion of registered prostitutes who during some period or periods of their career are infected with syphilis, it will be possible to form an approximate estimate of the number of policemen and surgeons requisite to constitute the vast force which would be necessary to accomplish the examinations with that frequency and on that extensive scale which the leading French hygienists insist on as indispensible in order to prevent registered women from being, as they are authoritatively affirmed to be now, "a perpetual source of contamination."

Dr. Armand Depres, Professor of the Faculty of Medicine of Paris, and many years surgeon to Lourcine Hospital in that city, condemns the system of compulsory medication of prostitutes now practiced in Paris as "illusory," and one of his reasons for condemning it he states as follows: "Because a woman can be registered without its being discovered that she has had the syphilis (for example, an invalid, scarcely out of my hands cured, and yet in whom the disease may reappear, and be transmitted, is at once and easily registered)." Referring to the system generally he says, "It is well that it should be stated that our system, with the Dispensary of the Prefecture of Police and the Prision of St. Lazare, achieves, by the way of remedy to this evil, *nothing* or *almost nothing*. . . . Unless all women who practice prostitution clandestinely can be superintended, how is it possible to arrest the propagation of syphilis."—*Compulsory Medication of Prostitutes by the State.*

